# Cast versus functional brace in the rehabilitation of patients with a rupture of the Achilles tendon: statistical analysis plan for the UK study of tendo Achilles rehabilitation (UK STAR) multi-centre randomised controlled trial

**DOI:** 10.1186/s13063-019-3380-x

**Published:** 2019-05-30

**Authors:** Ioana R. Marian, Matthew L. Costa, Susan J. Dutton

**Affiliations:** 1 Oxford Clinical Trials Research Unit, Centre for Statistics in Medicine, Nuffield Department of Orthopaedics, Rheumatology and Musculoskeletal Sciences, University of Oxford, Windmill Road, Oxford, OX3 7LD UK; 2 Oxford Trauma, Nuffield Department of Orthopaedics, Rheumatology, and Musculoskeletal Sciences, University of Oxford, The Kadoorie Centre, John Radcliffe Hospital, Oxford, OX3 9DU UK

**Keywords:** Rehabilitation, Achilles tendon, Rupture, Statistical analysis plan, Randomised controlled trial

## Abstract

**Background:**

The incidence of Achilles tendon rupture in the UK is increasing and the best rehabilitation strategy for patients treated non-operatively remains unclear. We describe a statistical analysis plan (SAP) for the UK study of tendo Achilles rehabilitation (UK STAR) multi-centre randomised trial.

**Methods/design:**

UK STAR is a 1:1, multi-centre, parallel, two-arm, superiority randomised controlled trial. This study aims to evaluate the use of functional bracing compared to plaster cast for the management of acute Achilles tendon rupture in adult patients treated non-operatively. The primary outcome is the Achilles Tendon Rupture Score measured at 9 months after injury and will be estimated based on a linear mixed effects regression model adjusted for the stratification factor (centre) and other key prognostic variables. Secondary outcomes include complications, quality of life and resource use evaluated at 8 weeks and at 3, 6 and 9 months after the injury. Missing data will be summarised and reported by treatment arm. Full details of the planned analysis methods are described in this paper. Further study design details are published in the UK STAR protocol.

**Discussion:**

The planned statistical analyses for UK STAR aim to reduce the risk of outcome reporting bias arising from prior data knowledge. Any changes or deviations from the current SAP will be described and justified in the final study report.

**Trial registration:**

International Standard Randomised Controlled Trial Number Registry, ISRCTN62639639. Registered on 22 June 2016.

## Background

Achilles tendon rupture affects over 11,000 people each year in the UK [1] with a detrimental impact on their walking and running. Achilles rupture can occur at any age, but most frequently affects male patients aged 30–39 years and increasingly female patients aged over 60 [1]. As more of the population remains active into older age, the UK incidence of achilles tendon rupture is increasing.

Several randomised controlled trials (RCTs) have evaluated the use of plaster cast and early movement and/or functional brace in the treatment of Achilles tendon rupture after surgical repair [2, 3]. For patients treated operatively, functional outcome, re-rupture rate and quality of life have been reported to be better among patients receiving functional bracing compared to those receiving plaster cast. Operative repair is associated with a reduced risk of re-rupture; however, it comes with an increased cost and risk of complications due to infection and wound healing [4]. Much research supports the use of functional brace as post-surgical treatment [5], however there is currently not enough evidence to suggest that functional bracing leads to better functional outcomes than plaster cast in patients choosing non-operative treatment [4]*.*

UK Study of tendo achilles rehabilitation (UK STAR) is a randomised controlled trial [6] aiming to compare functional outcomes between plaster cast and functional brace for patients with a rupture of the Achilles tendon that is managed non-operatively. This paper aims to report the details of the analysis plan (SAP) as agreed by the Data and Safety Monitoring Committee (DSMC) in September 2018 and has been prepared according to the published guidelines on the content of statistical analysis plans [7]. The trial is registered with the International Standard Randomised Controlled Trials database, reference number ISRCTN62639639.

## Methods and design

### Trial design

UK STAR is a pragmatic, multi-centre, parallel, two-arm, randomised controlled, superiority trial. Eligible patients are randomised 1:1, stratified by study centre using variable block sizes of 2, 4 and 6 in a 1:2:1 ratio via a secure, online randomisation system. Both plaster cast and functional bracing treatment techniques are routinely available at all recruiting sites. Stratification by centre helps to ensure any centre-related effect is equally distributed in the two trial arms. The patients and treating clinicians cannot be blind to the treatment as the type of rehabilitation used is clearly visible. The outcome data are collected and entered onto the trial central database from a paper form or via telephone by a research assistant/data clerk in the trial central office who is blinded to treatment arm to reduce the risk of assessment bias. Participants are also able to submit outcome data via an online questionnaire. Full details of the trial design, study population and study procedures have been published in the UK STAR protocol [6].

### Objectives

The primary objective is to quantify and draw inferences on observed differences in the Achilles Tendon Rupture Score (ATRS) [8] between the trial treatment groups at the 9-month follow up. The null hypothesis assumes that there is no difference in the ATRS outcome at 9 months between the two treatment arms.

Secondary objectives include:

- ▪ Quantifying and analysing observed differences in ATRS between the trial treatment groups at 8 weeks and at 3 and 6 months
- ▪ Identifying differences in health-related quality of life between the trial treatment groups in the first 9 months
- ▪ Determining the complication rate between the trial treatment groups in the first 9 months
- ▪ Investigating, using appropriate statistical and economic analytical methods, the resource use, costs and comparative cost-effectiveness in the trial treatment groups

### Outcomes

#### Primary outcome

The primary outcome is the ATRS measured at the 9-month follow up. The ATRS is a validated self-reported questionnaire [8, 9] consisting of 10 items to assess symptoms and physical activity specifically related to the Achilles tendon. Each item varies from 0 (major limitations/symptoms) to 10 (no limitations/symptoms) on an 11-point scale, measuring strength, fatigue, stiffness, pain, activities of daily living, walking on uneven surfaces, walking upstairs or uphill, running, jumping and physical labour. The ATRS score is derived from the sum of the 10 questions with a total score between 0 and 100. This value reaches a plateau between 6 and 9 months after rupture [8].

#### Secondary outcomes

The ATRS collected at 8 weeks and at 3-month and 6-month follow up is a secondary outcome. Other secondary outcomes include quality of life, complications and resource use:

- Quality of life is measured by the EuroQol 5 Dimensions 5 Levels (EQ-5D-5 L) [10], a validated, generic health-related questionnaire. This includes the EQ-5D-5 L descriptive system consisting of 5 dimensions, each with a 5-level answer possibility, which are converted to utility values, and the EQ visual analogue scale ( EQ VAS) health thermometer scale, with values between 0 and 100, where 100 represents the best imaginable health. EQ-5D is reported at the 8-week and the 3-month, 6-month and 9-month follow up.
- Complications include re-rupture, blood clots/emboli, pressure areas and neurological symptoms in the foot. These are recorded from medical notes at the 8-week review when the cast/brace is removed and are patient-reported at 3, 6 and 9 months.
- The resource use data are collected at 8 weeks and at 3, 6 and 9 months and will be used to conduct a prospective health economic evaluation in the two trial arms.

### Sample size

The sample size calculation required 330 participants to be randomised. This assumed a population standard deviation (SD) of 20 points for the primary outcome ATRS at 9 months after injury [11] and a minimum clinically important difference (MCID) of 8 [9]. At an individual patient level, a difference of 8 points represents the ability to walk upstairs or run with “some difficulty” versus with “great difficulty”; at a population level, 8 points represents the difference between a “healthy patient” and a “patient with a minor disability” [9]. Given the SD assumption, a 5% two-sided significance test with 90% power to detect an MCID of 8 points led to a requirement of 264 participants to be randomised. One aspect of non-compliance was expected to be crossover between interventions. This assumed a 20% loss of primary outcome data due to patients crossing over and those lost to follow up, based on previous experience of similar trials within our unit.

The trial reached its primary recruitment target of 330 participants prior to the end of the planned recruitment period, but recruitment was extended after consultation with the DSMC and Trial Steering Committee (TSC). The sample size was recalculated based on a larger population variability equivalent to 25 points SD, MCID 8 and 20% loss to follow up, which led to 516 participants, with a maximum limit set at 550 participants, to be recruited into the trial.

### Statistical analysis

#### General analysis principles

A blinded analysis of data (not separated by treatment arm) will be undertaken prior to the final data lock in order to assess the distribution of variables, missing data distributions and outliers. This analysis will also be used to confirm the pre-specified key prognostic variables (centre, age, gender, baseline ATRS) to be included in the adjusted analysis and will also explore additional prognostic variables to be included in further supporting analyses. These will include smoking, diabetes mellitus and time since injury. The SAP will be updated following the blinded analysis and approved prior to the final data lock when the treatment code will be added to the database.

The primary analysis population will be intention to treat (ITT) and a complier average causal effect (CACE) population will be used as supporting analyses. The ITT population will include all randomised participants in their allocated groups and the CACE population will consist of randomised participants compliant with the intervention [12]. Patients will be considered compliant with the intervention if they receive the treatment allocated at randomisation and continue to wear it for a minimum period of 6 weeks. Primary outcome ATRS and secondary outcome EQ-5D-5 L will be analysed in the ITT and CACE populations, and complications will be analysed in the ITT population. Sensitivity analyses will be conducted to explore the definition of compliance with treatment and missing data assumptions. Tests will be two sided and considered to provide evidence for a significant difference if *p* values to three decimal places are less than 0.05 (5% significance level).

#### Descriptive analyses

**Fig. 1:**
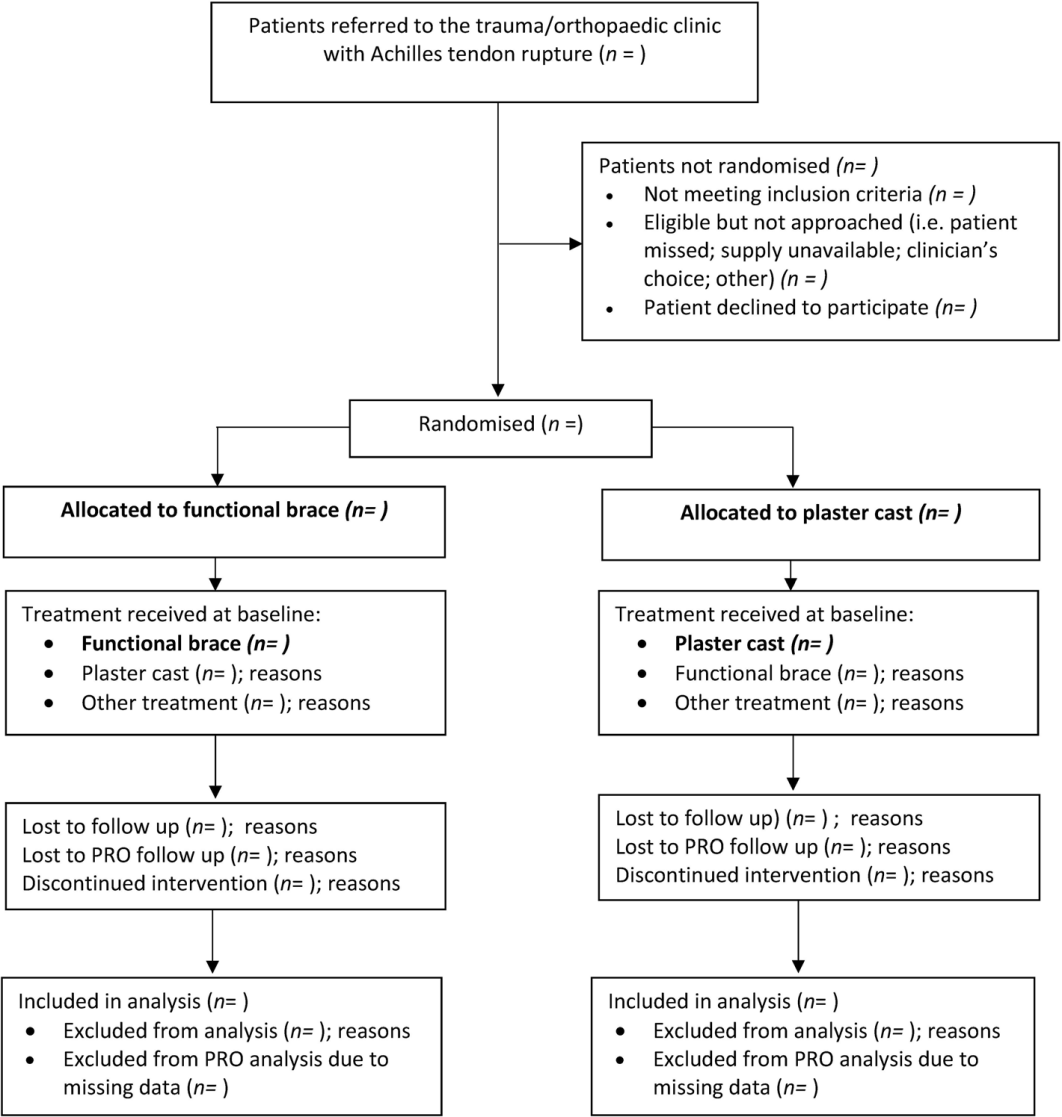
CONSORT flow diagram template (based on CONSORT PRO extension)

A Consolidated Standards of Reporting Trials for Patient Reported Outcomes [13] (CONSORT PRO extension) flow chart will be used to report the number of participants at each stage of the trial (see Fig. 1). Participant baseline characteristics will be reported by treatment group and overall. These will be presented as numbers with percentages for categorical variables, and either means and standard deviations or medians with interquartile range (IQR) for continuous variables. There will be no tests of statistical significance nor confidence intervals (CIs)for differences between randomised groups in any baseline variable.

Baseline characteristics will include stratification factors and the following variables:

- Gender
- Age
- Days since injury; mechanism of injury; side of injury
- Height; weight; body mass Index (BMI)
- Smoking; cigarettes per day; number of years smoking
- Alcohol consumption
- Medications; medical conditions, including diabetes mellitus
- ATRS before injury
- EQ-5D-5 L before injury and baseline after injury
- Centre

#### Withdrawals from treatment and/or follow up

Withdrawals and losses to follow up, together with reasons, will be reported by treatment arm and compared in each of the periods leading up to the 8-week and 3-month, 6-month and 9-month time point. In the event of a tendon re-rupture following which the participant undergoes surgery, the participant will not be treated as lost to follow up. Differential losses between the groups will be compared and deaths (and their causes) will be reported separately.

#### Missing data

The patterns of data availability for primary and secondary outcomes from baseline to end of follow up and reasons for missingness, where known, will be summarised in the two treatment groups (functional brace and plaster cast). The nature and pattern of missing data (missing completely at random (MCAR), missing at random (MAR) or missing not at random (MNAR)) will be explored.

The primary outcome ATRS is expected to have low levels of item missingness. Validation rules will ensure that data are entered in the correct format, within valid ranges, minimising the chance of missing data. Where ATRS items are missing and a minimum of 5 out of 10 ATRS items are present, a pro-rata estimation of the ATRS score will be imputed based on the average (mean or median values, depending on the data distribution in the overall group) of the available ATRS item responses for the primary analysis. The extent of pro-rata estimation will be reported for each treatment group.

Missing continuous primary and secondary outcomes will be handled as part of the likelihood-based estimation of the linear mixed model in the primary analysis where the MAR assumption holds, allowing sufficient generality to include baseline variables thought to be important predictors [14]. Sensitivity analyses will be undertaken to explore departures from this assumption if applicable, using multiple imputation (MI).

#### Compliance

One group of patients will receive a functional brace (walking boot) after randomisation and one group will receive a plaster cast. The compliance with treatment will be reported by intervention group and summarised where possible with reasons for not receiving the assigned treatment. A CACE analysis [12] will be used to estimate the impact of treatment compliance on primary and secondary outcomes based on an instrumental variable approach. The association between compliance and treatment group will be evaluated. Sensitivity will be analysed for the defined compliance period (compliant with treatment for 6 weeks or more) in the populations compliant with treatment for 2 weeks or more and 4 weeks or more.

#### Analysis of primary outcome

The primary outcome, ATRS at 9 months after randomization, will be summarised on an ITT basis as mean and SD in each of the two treatment groups, functional brace and plaster cast, assuming the data are approximatively normally distributed. The main findings of the trial will be reported as the difference in the ATRS between the two treatment arms, estimated with a linear mixed effects regression model, including outcome information from all follow-up points and adjusting for centre, age, gender and baseline ATRS. Supporting analyses will also be reported, including other important prognostic variables as identified during the blind review. As individual clinicians will treat only a small number of patients, important clinician-specific effects are not expected, but recruiting centre will be included in the model as a random effect factor to adjust for potential cluster differences. Estimates of treatment effects will be presented with 95% CIs.

Where severe departure from normality in the difference in ATRS between the two groups is identified (i.e. by checking residuals), the first approach to attain normality will be data transformation or the use of a different metric such as change from baseline. If the data cannot be transformed to reflect normality, then the Mann-Whitney U test will be used (in this case, no further adjustments will be made) and the medians and IQRs will be reported in each treatment arm.

Differences between treatment groups will be analysed unadjusted using the Student *t* test, based on a normal approximation for the ATRS score. Estimates of treatment effects will be presented with 95% CIs for both adjusted and unadjusted analyses. The ITT adjusted analysis of the primary outcome will be used to determine the success or otherwise of the trial. Sensitivity analysis to confirm the robustness of the ITT primary analysis conclusions will be repeated in the CACE population and using multiple imputation, where appropriate.

To explore recovery in the two treatment groups over time, the ATRS will be analysed further. This will summarise the longitudinal data collected at all four time points to a single value, the area under the curve (AUC) [15] and will facilitate comparison of the ATRS between treatment groups over time. Parameter estimates from the mixed effects models will be used to calculate AUCs for each treatment group from baseline to the 9-month follow up. This will provide an overall estimate of recovery over time in each group. Larger ATRS scores are associated with fewer limitations/difficulties related to the injured Achilles tendon, therefore larger AUCs will suggest improved function. AUCs for each treatment group and their difference calculated using the *t* test will be presented together with their associated 95% CI. The EQ-5D-5 L will also be analysed in this way.

#### Analysis of secondary outcomes

Continuous secondary outcomes will be analysed on an ITT basis using the methodology described for the primary outcome, and results will be reported at each intervening time point. Complications in each treatment arm will be explored and analysed on an ITT basis, reported as numbers (with percentages) and compared at 8 weeks and at 3, 6 and 9 months using Fisher’s exact or chi-squared test. Results will be reported with their associated 95% CI and *p* values for comparison between the two treatment groups.

#### Safety

Some adverse events and serious adverse events (SAEs) are foreseeable as part of the proposed treatment and are recorded as complications. Unexpected but related SAEs and suspected unexpected serious adverse reactions (SUSARs) that occur between date of consent and the 9-month follow-up point will be reported and compared between the functional bracing and plaster cast groups in the ITT population by examination of 95% confidence intervals for differences in incidence.

### Subgroup analysis

There are no planned subgroup analyses for any of the follow-up primary or secondary outcomes.

### Health economic analysis

A separate heath economic and cost-effectiveness analysis will be conducted and details of this analysis described in a separate health economics analysis plan.

### Statistical packages

All analysis will be carried out using appropriate validated statistical software such as STATA, SAS, SPLUS or R. The relevant package and version number will be recorded in the statistical report.

## Discussion

The UK STAR trial will provide functional outcome data on two rehabilitation strategies, functional brace versus plaster cast, in non-operatively managed patients with a rupture of the Achilles tendon. One study limitation is that it will not be possible to blind patients to their allocated treatment, as the plaster cast or functional brace will be clearly visible.

This paper provides details of the planned statistical analyses for UK STAR and any changes from the current protocol or SAP will be described and justified in the final report. The aim of the pre-specification of the analysis is to reduce the risk of outcome reporting bias and data-driven results [16].

## Trial status

Recruitment into the trial opened on 1 August 2016 and closed on 31 May 2018. A total 541 participants were recruited from 39 sites. Follow up for the trial outcome data will be completed by March 2019, which will then be followed by the analysis.

## Abbreviations

ATRS: Achilles Tendon Rupture Score
AUC: Area under the curve
BMI: Body Mass Index
CACE: Complier average causal effect
CI: Confidence interval
CONSORT PRO: Consolidated Standards of Reporting Trials for Patient Reported Outcomes
DSMC: Data and Safety Monitoring Committee
EQ VAS: Euroqol visual analogue scale
EQ-5D-5L: EurQol 5 Dimensions 5 Levels
HTA: Health technology assessment
IQR: Interquartile range
ISRCTN: International Standard Randomised Controlled Trial Number
ITT: Intention to treat
MAR: Missing at random
MCAR: Missing completely at random
MCID: Minimum clinically important difference
MI: Multiple imputation
MNAR: Missing not at random
NHS: National Health Service
NIHR: National Institute for Health Research
OCTRU: Oxford Clinical Trials Research Unit
RCT: Randomised controlled trial
SAE: Serious adverse events
SAP: Statistical analysis plan
SD: Standard deviation
SUSAR: Suspected unexpected serious adverse reactions
TMG: Trial Management Team
TSC: Trail Steering Committee
UK STAR: UK Study of tendo achilles rehabilitation
